# Supplementary material for: Maternal and Intrauterine Influences on Feto-Placental Growth Are Accompanied by Sexually Dimorphic Changes in Placental Mitochondrial Respiration, and Metabolic Signalling Pathways
Source: Cells. 2023 Mar 3;12(5):797. doi: 10.3390/cells12050797 (PMC10000946; doi:10.3390/cells12050797)
Supplement: Supplementary file 1 [file cells-12-00797-s001.zip › cells-2208897-supplementary.pdf]

cells

MDPI

Supplementary Materials

Maternal and Intrauterine Influences on Feto-Placental Growth Are Accompanied by Sexually Dimorphic Changes in Placental Mitochondrial Respiration, and Metabolic Signalling Pathways

Esteban Salazar-Petres [1,†], Daniela Pereira-Carvalho [2,†], Jorge Lopez-Tello [2,*,‡] and Amanda N. Sferruzzi-Perri [2,*,‡]

1 Departamento de Ciencias Básicas, Facultad de Ciencias, Universidad Santo Tomás, Valdivia 5090000, Chile; e.salazar.petres@gmail.com
2 Centre for Trophoblast Research, Department of Physiology, Development and Neuroscience, University of Cambridge, Cambridge CB2 3EG, UK; carvalho.pdaniela@gmail.com
* Correspondence: jl898@cam.ac.uk (J.L.-T.); ans48@cam.ac.uk (A.N.S.-P.)
† These authors contributed equally to this work.
‡ These authors contributed equally to this work.

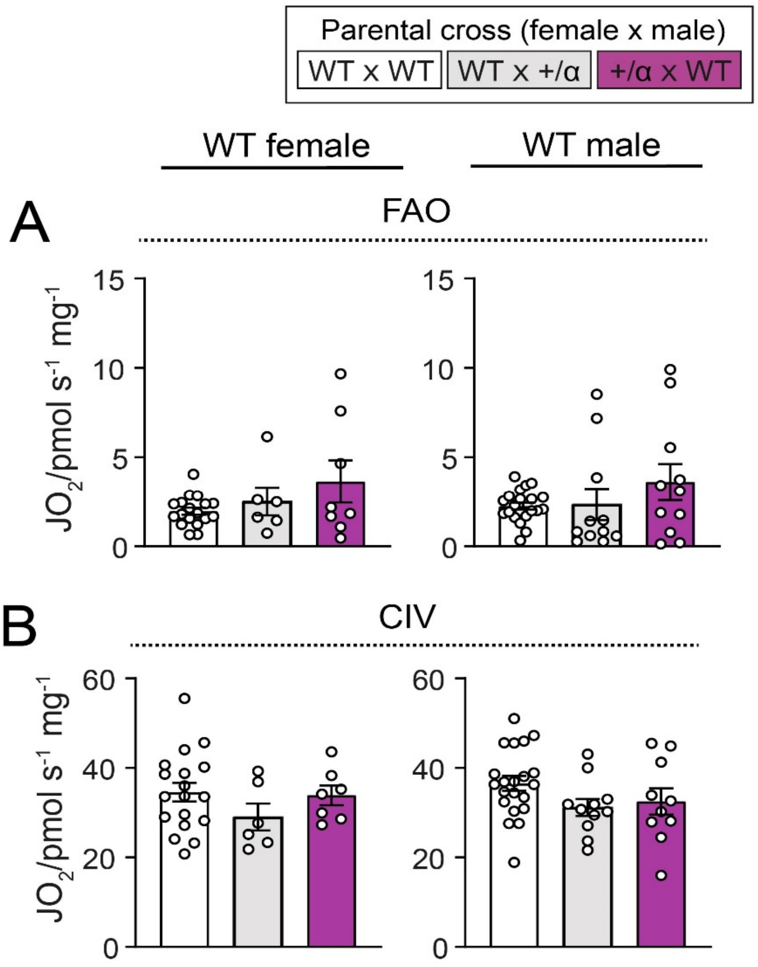

Figure S1. Placental mitochondrial bioenergetics of WT conceptuses in response to littermate and/or maternal p110*α* deficiency. Oxygen consumption linked to mitochondrial fatty acid oxidation (**A**) and CIV (**B**) in the placental LZ of females and males on day 18 of pregnancy. Data are from WT fetuses generated by WT x WT, WT x *α*/+ and *α*/+ x WT parental crosses (n = 1–2 fetuses/sex/dam

with 5–12 dams/group) and are displayed as individual data points with mean ± S.E.M. Data were analyzed by one-way ANOVA with Tukey *post hoc* pairwise comparisons.

Table S1. List of primary antibodies used in this study.

Primary Antibody	Host/ Isotype	Manufacturer, Catalogue Number	Dilution
**AKT**	Rabbit	Cell Signalling, 9272	1/1000
**Phospho-AKT (Ser473)**	Rabbit	Cell Signalling, 9271	1/1000
**Phospho-AMPK (Thr172)**	Rabbit	Cell Signalling, 2535	1/1000
**P44/42 MAPK (Erk1/2)**	Rabbit,	Cell Signalling, 4695	1/1000
**Phospo-MAPK-p44/42 (Erk1/2; Thr202/Tyr204)**	Rabbit IgG	Cell Signalling, 4370	1/1000
**Total-p38 MAPK**	Rabbit IgG	Cell Signalling, 8690	1/1000
**Phospho-p38 MAPK (Thr180/Tyr182)**	Rabbit IgG	Cell Signalling, 4511	1/1000
**UCP2**	Rabbit IgG	Biolegend, 615902	1/500
**PGC-1α**	Rabbit	Santa Cruz, sc-13067	1/1000
**OXPHOS**	Mouse IgG	Thermo Fisher, 45-8099	1/250
**Citrate synthase**	Rabbit	Abcam, ab9660	1/1000
**PPAR-γ**	Mouse IgG	Santa Cruz, sc-7273	1/200

Table S2. Litter size and composition.

	WT x WT	WT x α/+	α/+ x WT	p Value
**Litter size**	7.92 ± 0.36	7.40 ± 0.93	7.46 ± 0.49	0.7251
**% Females in the litter**	48.28 ± 3.46	44.33 ± 6.30	59.44 ± 6.25	0.1474
**% Mutants in the litter**	-	41.43 ± 11.01	61.33 ± 6.30	0.1933
**% Mutant females in the litter**	-	32.02 ± 10.36	35.12 ± 7.29	0.1234
